# Telepsychiatry in Oman within the broader framework of telehealth

**DOI:** 10.1192/bji.2025.10048

**Published:** 2026-08

**Authors:** Tamadhir Al-Mahrouqi, Alya Al Harrasi, Fatma Al Rajhi, Hamed Al Sinawi, Mohammed Al-Alawi

**Affiliations:** 1 Psychiatrist, Department of Behavioral Medicine, Sultan Qaboos University Hospital, Muscat, Oman. Email: t.almahrouqi@squ.edu.om; 2 Psychologist, Department of Behavioral Medicine, Sultan Qaboos University Hospital, Muscat, Oman; 3 Doctor, General Foundation Program, Oman Medical Specialty Board, Muscat, Oman; 4 Senior Consultant Psychiatrist, Department of Behavioral Medicine, Sultan Qaboos University Hospital, Muscat, Oman; 5 Consultant Psychiatrist, College of Medicine and Health Sciences, Sultan Qaboos University, Muscat, Oman

**Keywords:** eHealth, mHealth, telehealth, telemedicine, telepsychiatry

## Abstract

In recent years, the Sultanate of Oman has made strides in telehealth services to improve healthcare access for underserved patients, specifically through telephone consultations and digital appointment scheduling. However, the lack of technological infrastructure, insufficient training for healthcare providers and the absence of a unified digital system limit its full potential. As a result, patients in remote areas experience delays in diagnosis and treatment.

Telehealth, a subset of eHealth, refers to the comprehensive use of telecommunications technology in healthcare.^1^ It enables interactions between patients and providers through email, telephone, video-conferences and the internet.^1^ Telemedicine falls under the broader scope of telehealth but focuses specifically on clinical services.^1^ Telehealth and telemedicine both cover a range of services, such as medical education, remote patient monitoring, virtual consultations, wireless health applications, and the transmission of medical images and reports.^1^ Telehealth technologies are being widely implemented as an effective and affordable way to deliver and access quality healthcare services, especially for patients in rural or underserved areas.^1^ Additionally, telehealth improves efficiency without additional costs, reduces patient travel and waiting times and maintains quality of care, increasing patient satisfaction.^1^ Given the promising rise of telehealth services in the Middle East, there is a growing need to identify best practices for their successful implementation.

## Objective of the article

The objective of the article is to examine the current state of telepsychiatry in Oman within the broader framework of telehealth, highlighting key challenges, initiatives and future directions for improving digital mental healthcare services. To the best of our knowledge, this is the first review that covers all telehealth services and policies in Oman.

## Healthcare in Oman

The Sultanate of Oman is one of the Eastern Mediterranean Region countries.^2^ As of February 2025, the total population of Oman is around 5.2 million (57% Omani, 43% Non-Omani).^2^ Oman’s Ministry of Health (MoH) provides most of the country’s medical services and is accessible free of charge to its citizens.^3^ The overall number of medical doctors employed by the MoH is around 6500, and a further 2800 doctors are employed by the public sector.^2^ However, the number of doctors has decreased from 21 per 10 000 population in 2018 to 19.1 per 10 000 population in 2022.^2^ Regarding Oman’s human resources in mental healthcare, there are 5.8 psychiatric beds, 1.54 psychiatrists and 10.3 nurses per 100 000 population.^4^ In 2022, there were a total of 108 838 visits to MoH psychiatric clinics, 15 162 of which were new cases, and most patients were between 19 and 59 years of age.

## The emergence of telehealth in Oman

Despite 4.58 million internet users in Oman in 2024,^5^ access to telehealth services remains limited. The country is working towards the government’s Oman Vision 2040 programme, intending to incorporate digital technology to improve and facilitate the delivery of healthcare services.^6^ In 2021, the MoH launched a newer version of Shifa, a digital platform, to improve the healthcare delivery experience.^6^ The app offers several electronic services, including access to personal information, medical reports and appointments.^6^ Similarly, the Oman Medical Specialty Board has established a Simulation and Innovation Center to enhance the use of medical simulation techniques and modern technologies in specialised medical training. In tandem with such initiatives, academics have been dedicating efforts to creating solid ground for establishing a virtual healthcare system.^7^

## Current implementation of telehealth services

### Sultan Qaboos University Hospital initiatives

The use of telehealth has vastly increased in Oman since the onset of the COVID-19 pandemic. At the forefront of telehealth is Sultan Qaboos University Hospital (SQUH), a leading tertiary care teaching hospital based in the capital of Oman, Muscat. During the pandemic lockdown, the Department of Child Health at SQUH launched virtual clinic services to continue providing care for out-patients.^8^ Surprisingly, patients were comfortable using video-conferencing and requested the continued use of this system even post-pandemic.^8^ Meanwhile, the Department of Urology at SQUH reduced the number of out-patient clinic visits by offering telephone consultations and allowing online prescription refills.^9^ Previously, SQUH had allowed patients to refill their prescriptions by sending a message through the WhatsApp messaging service, with their medical record number and clinic name without the need for in-person visits.^9^ However, this service was discontinued at the start of 2025.

### Ministry of Health initiatives

The largest tertiary hospital in Muscat is the Royal Hospital, which offers telephone consultations for patients with chronic diseases or those living in remote areas. In primary care, family physicians have found phone consultations beneficial specifically for patients with chronic conditions in terms of avoiding the risk of infection.^10^ However, there were technical support difficulties, limited staff training on telephone consultations and concerns about patient privacy.^10^ A study interviewing psychiatrists at Al Masarra Hospital, a tertiary care psychiatric facility, in Muscat revealed that telephone consultations offered convenience and flexibility to manage non-clinical responsibilities and provide tailored support. They also benefitted patients by improving appointment adherence, reducing stigma and providing financial savings compared with in-person consultations.^11^

## The role of telepsychiatry in Oman’s mental healthcare

A growing number of studies evaluating the effectiveness of telepsychiatry in Oman have reported an overall high satisfaction rate among both patients and healthcare providers towards this promising service. A randomised controlled trial tested the effectiveness of online therapy for Omani patients experiencing anxiety and depression due to COVID-19.^12^ Online therapist-guided therapy was found to be superior to internet-based self-help therapy in reducing levels of anxiety and depression.^12^ Building on this study, a qualitative exploration was conducted to understand patients’ and therapists’ experiences of video-conferencing therapy sessions.^7^ The patients appreciated the flexible scheduling, reduced travel time and cost, and the lowered stigma around mental healthcare.^7^ The therapists found telemental health beneficial in reducing the risk of intrahospital infection, lowering healthcare costs and achieving a better work–life balance.^7^

## Challenges and barriers to telemedicine and telepsychiatry implementation

A growing concern in several countries, including Oman and Qatar, is the rapid utilisation of this technology without overarching legal oversight.^13^ Common barriers are found in Arab countries, such as insufficient knowledge among healthcare workers, inadequate patient participation, insufficient technological infrastructure and concerns regarding financial expenses. Specifically in Gulf Cooperation Council (GCC) countries, the top challenges found are cultural, technological, legal and regulatory.

A study exploring the efficiency of telepsychiatry using phone consultations in Oman has highlighted confidentiality concerns, the absence of visual cues and the shortage of trained staff as the main barriers to implementing this service.^11^ There were also technological issues, such as the unavailability of a shared network for patient data, which limited communication between care providers in different institutions and thus affected the continuity of care. Psychiatrists reported that insufficient phone lines and inadequate financial support limited the number of telepsychiatry clinics and compromising their running. They also revealed that most patient records are outdated and therefore time was wasted trying to get the correct contact information, which often led to delays in appointments. Similarly, the lack of electronic prescriptions and insufficient access to child and adolescent psychiatric medications at the primary and secondary services caused burden on patients and their families. Although their consultations are conducted remotely, they still need to return to the tertiary care facility, Al Masarra Hospital, to collect their medications, impairing the intended convenience provided by telepsychiatry. Beyond technical constraints, the study reported difficulties in verifying patient identity, performing physical/neurological assessments and mental status examinations, and having access to a private environment at home necessary for the call. This has also raised worries about breaches of patients’ personal data. Additionally, it mentioned safety doubts regarding the remote management of high-risk conditions in virtual settings owing to the lack of established regulations.

A big concern is the lack of clear protocols and law enforcement in managing psychiatric emergencies that might arise during remote consultations. Patients with suicidal or homicidal risk will need prompt evaluation and in-patient care, but psychiatrists discussed difficulty in reporting those incidents and making sure the patient can reach an emergency room without an established support system.^11^

Telepsychiatry requires the provider to be trained in the use of digital platforms and how to manage technical difficulties, and to have high in social intelligence to recognise verbal and visual cues that aid in establishing accurate diagnosis.^7^ Healthcare workers who were not offered proper training in telepsychiatry complained about increased stress and the struggle to manage crises, which compromises the benefits of this service.^7^

## Regulatory framework and policy considerations for telehealth

In 2020, the MoH issued its first clinical guidelines for the use of telehealth in Oman healthcare facilities.^14^ These guidelines mandate healthcare institutions and practitioners to obtain MoH licences, conduct consultations in well-equipped facilities and use secure technology to protect patient data. The guidelines address patients’ consent and the confidentiality of information. All patient data must be securely stored and made readily accessible in the event of patient transfer to another healthcare facility. The aim is to ensure that telehealth services maintain the same quality standards as in-person care. An assessment must ensure that the patient’s clinical condition is suitable for telehealth services before proceeding with remote consultations. In addition, the telehealth licence is valid for a period of 2 years and must be renewed 30 days prior to its expiration to continue practising telehealth services.

Neighbouring GCC countries have implemented similar telehealth policies and regulations, but within a more detailed and publicly accessible framework. For instance, the United Arab Emirates (UAE) introduced telehealth services in 2013 and significantly expanded them in 2019 by establishing the right for every citizen to access a doctor 24/7 through digital apps.^15^ The UAE was also the first to lead on telemedicine intensive care unit (tele-ICU) services for remote critical care and it offered a wide range of virtual clinics and specialty services during the COVID-19 pandemic.^15^ Qatar’s telehealth services have existed since 2016, but it definitely upscaled those services in response to the pandemic, for example by introducing an urgent consultation telemedicine service, which provided 80% of regular out-patient services.^13^

Regarding regulatory safeguards, several countries in the region have thoughtfully implemented patient safety in telehealth. For example, Saudi Arabia allows credentialled healthcare professionals, such as nurses, to provide telehealth services related to diagnosis and treatment only under the direction and clinical supervision of the treating physician.^16^ Additionally, Saudi Arabia requires health insurance to cover telehealth services.^16^ The UAE regulations prohibit the prescription of any narcotic, controlled or semi-controlled medications via telehealth.^17^ Although several Gulf countries have clear and detailed protocols for managing patients with severe or high-risk conditions via telehealth, Oman has yet to implement similar protocols. The establishment of clear telehealth guidelines that are accessible to both healthcare workers and the public would support Oman in achieving high-quality remote care.

## Future directions for telehealth in Oman

Looking forward, the Oman Vision 2040 programme has aligned with the evolution of technology by setting goals of adopting modern medical advances in healthcare, emphasising the crucial role of digitalisation in improving the quality of care.^6^ The vision lists the integration of virtual clinics as one of its specific areas of focus and aims for their introduction in three tertiary hospitals in Muscat. In addition, the MoH plans to integrate artificial intelligence technologies for the early detection of breast cancer in five healthcare institutions.

Taking into consideration the barriers investigated by researchers, several further recommendations can be considered. A key recommendation would be the development of a unified electronic healthcare system linking primary, secondary and tertiary care, thus ensuring the accuracy of data transmission and the facilitation of physical examinations and necessary laboratory and imaging investigations at primary and secondary healthcare centres. This shared electronic platform would link patients, general practitioners and family physicians in rural areas with psychiatrists in subspecialised tertiary care in Muscat, which would cut unnecessary travel costs and increase the quality of patient care. With the establishment of such a shared system, more research opportunities would arise to investigate the gaps filled by bridging care services. Another area of improvement is creating clear guidelines about the use of telecommunication in healthcare, addressing issues of conduct and setting rules for directing patients to the next level of care in emergencies. This should be in line with directed training programmes for each medical specialty, completion of which earns a licence to practice telehealth.

Moreover, the MoH in Oman should follow the lead of countries that are ahead in this regard and provide set regulations and an officially accredited telemedicine training programme. A good example is Saudi Arabia, which introduced a telemedicine licensing regimen in 2018, marking a stepping stone into the development and legalisation of the sector.^18^ The official adoption of national telemedicine regulations was followed by the launch of telemedicine centres that provided facilities equipped to practise telemedicine and conducted training programmes in accordance with the regulations. The establishment of these telemedicine centres has been opened to foreign investors, which has attracted international expertise to grow the field further.^18^ One promising approach is investing in video-conferencing, which offers better advantages over phone consultation by allowing providers to observe essential visual cues. A controlled trial providing video-conferenced guided therapy has reported favourable outcomes.^12^ With the remarkable advancements in technology, services like virtual reality can further aid in creating an engaging environment that would enhance the patient and physician experience. Likewise, artificial intelligence could improve telemedicine by offering advanced data analysis to help in diagnosis and treatment. It would also be a valuable tool in automating administrative tasks like documentation and appointment scheduling. These advancements will help bridge existing gaps and enhance the overall efficiency of healthcare services in Oman.

## Conclusion

Telehealth in Oman is an evolving healthcare model that promises to optimise the quality of patient care. Factors like convenience, easier rearrangements, better time management and cost-effectiveness draw wider acceptance of digital health services. It is also anticipated that such services will improve continuity of care, especially by connecting services in rural areas with centralised tertiary hospitals. The efficiency of telehealth could be expanded by addressing technological, organisational and financial restrictions, setting room for further improvement.
